# Hypermethylated *PCDHGB7* as a universal cancer only marker and its application in early cervical cancer screening

**DOI:** 10.1002/ctm2.457

**Published:** 2021-06-20

**Authors:** Shihua Dong, Qi Lu, Peng Xu, Limei Chen, Xiaoling Duan, Zhanrui Mao, Baolong Zhang, Long Sui, Yudong Wang, Wenqiang Yu

**Affiliations:** ^1^ Research and Development Department Shanghai Epiprobe Bio‐Technology Co., Ltd Shanghai China; ^2^ Shanghai Public Health Clinical Center Department of General Surgery, Huashan Hospital Cancer Metastasis Institute Laboratory of RNA Epigenetics, Institutes of Biomedical Sciences Shanghai Medical College, Fudan University Shanghai China; ^3^ Department of Obstetrics and Gynecology Jinshan Hospital of Fudan University Shanghai China; ^4^ Department of Gynecology, International Peace Maternity and Child Health Hospital Shanghai Jiaotong University School of Medicine Shanghai China; ^5^ Medical Center for Diagnosis and Treatment of Cervical Disease, Obstetrics and Gynecology Hospital, Fudan University Shanghai Key Laboratory of Female Reproductive Endocrine‐Related Diseases Shanghai China

**Dear Editor**,

We identified hypermethylated *PCDHGB7* as a novel cancer marker and applied it to early cervical cancer (CC) screening. It outperforms the widely implemented high‐risk human papillomavirus (hrHPV) test and ThinPrep cytologic test (TCT) and even can be used in the self‐sampled vaginal secretions, proving itself as a much more convenient yet highly effective screening method.

DNA methylation aberration occurs during cancer progression. DNA methylation has emerged as a promising diagnostic, prognostic, and predictive biomarker of various types of cancer.^1^ However, the common biomarker of cancers has been rarely explored. Previously, we provided the concept of Universal Cancer Only Marker (UCOM) and identified hypermethylated *HIST1H4F* as the first UCOM marker.^2^ In our genome‐wide methylation analysis, we found PCDH family genes were cancer cell‐differentially methylated genes (CC‐DMG).^2^ In the current study, we focused on *PCDHGB7*, a member of the protocadherin gamma gene cluster, which plays critical roles in the establishment and function of specific neuronal connections,^3^ and investigated whether it could be a novel UCOM marker. As CC is one of the most common female malignancies^4^ and the widely implemented hrHPV and TCT yield a high false‐positive rate,^5^, ^6^ we aimed to applied *PCDHGB7* in the early CC screening.

We compared the methylation status of *PCDHGB7* in 17 cancer types with their corresponding normal tissues in TCGA and GEO database (*n* = 7114). It turned out *PCDHGB7* was hypermethylated in all cancer types (Figure 1A). When analyzing FIGO staging, we found that *PCDHGB7* was already hypermethylated in stage I of all cancer types analyzed (Figure S1), suggesting hypermethylated *PCDHGB7* could be an early‐stage cancer indicator. Additionally, in different histological types, keratinizing squamous cell carcinoma, lymphovascular invasion, or histologic grades, there was no methylation difference of *PCDHGB7* (Figure S2). To verify these analytical results, we collected 13 types of clinical cancer samples (*n* = 727), in which *PCDHGB7* was hypermethylated accordingly (Figure 1B). Hypermethylation may account for the downregulated expression of *PCDHGB7* (Figure S3) and the lower frequency of CTCF peaks located on *PCDHGB7* promoter (Figure S4). Additionally, we assessed the performance of *PCDHGB7* hypermethylation as a biomarker for distinguishing between cancer and normal samples. The area under the curve (AUC) values were obtained for distinguishing 13 types of clinical cancer and control tissues with pyrosequencing data (Figure 1C and Table S1). It showed that all the AUC was larger than 0.85 (Table S1), especially in biliary cancer (AUC = 0.98) and esophagus cancer (AUC = 0.99). These results highly suggested that hypermethylated *PCDHGB7* can serve as a novel UCOM marker and play vital roles in CC progression.

**FIGURE 1:**
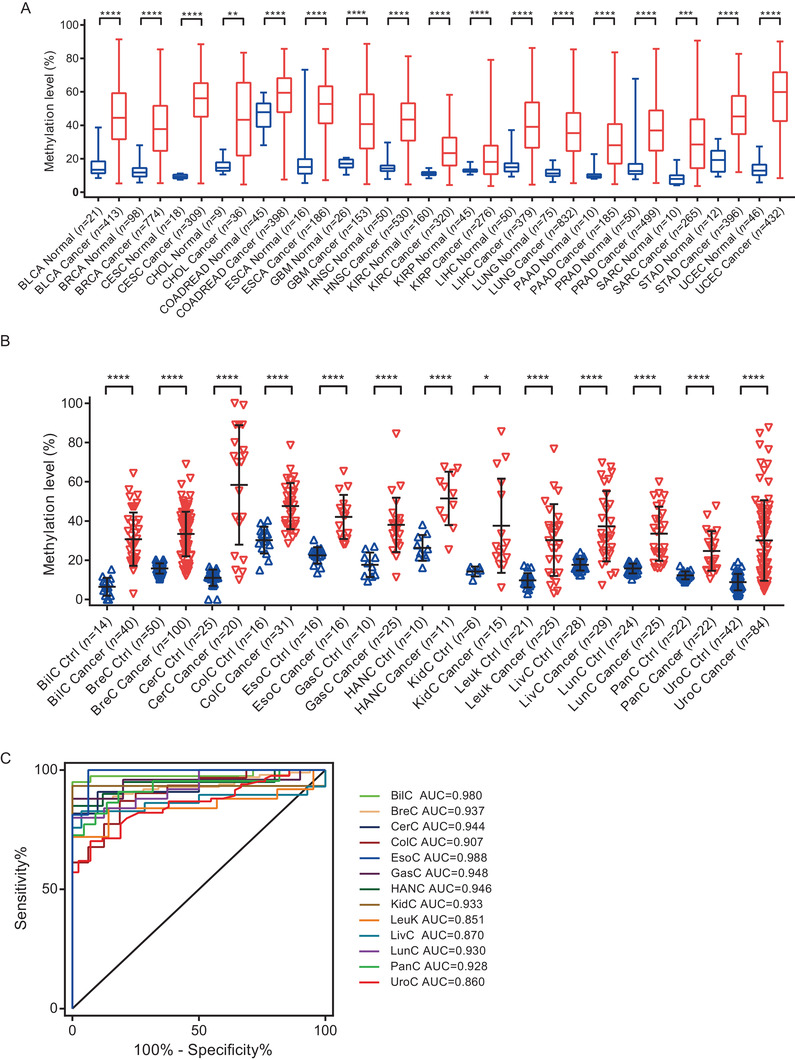
Hypermethylated *PCDHG*B7 is identified as a UCOM marker. (A) *PCDHGB7* was hypermethylated in 17 cancer types compared with their normal tissues in TCGA databases. Box and whiskers plots were plotted; box represents the upper quartile, lower quartile, and median; whiskers represent minimum to maximum. BLCA, bladder urothelial carcinoma; BRCA, breast invasive carcinoma; CESC, cervical squamous cell carcinoma and endocervical adenocarcinoma; CHOL, cholangiocarcinoma; COADREAD, colon adenocarcinoma and rectal adenocarcinoma; ESCA, esophageal carcinoma; GBM, glioblastoma multiforme; HNSC, head and neck squamous cell carcinoma; KIRC, kidney renal clear cell carcinoma; KIRP, kidney renal papillary cell carcinoma; LIHC, liver hepatocellular carcinoma; LUAD‐LUSC, lung adenocarcinoma and lung squamous cell carcinoma; PAAD, pancreatic adenocarcinoma; PRAD, prostate adenocarcinoma; SARC, sarcoma; STAD, stomach adenocarcinoma; UCEC, uterine corpus endometrial carcinoma. (B) *PCDHGB7* hypermethylated was confirmed in 13 types of cancers compared with their normal tissues in clinical samples. Error bar represents upper quartile, lower quartile, and median. (C) The AUC values for distinguishing cancer from control tissues in 13 cancer types. BilC, biliary cancer; BreC, breast cancer; CerC, cervical cancer; ColC, colorectal cancer; EsoC, esophagus cancer; GasC, gastric cancer; HANC, head and neck cancer; KidC, kidney cancer; Leuk, leukemia; LivC, liver cancer; LunC, lung cancer; PanC, pancreatic cancer; UroC, urothelial cancer. In both (A) and (B), *P* values were calculated using the two‐tailed unpaired parametric test by GraphPad Prism 7.0. *, *P* < 0.05; **, *P* < 0.01; ***, *P* < 0.001; ****, *P* < 0.0001

The management strategies for high‐ and low‐grade squamous intraepithelial lesion (HSIL, LSIL) are distinct; hence, there is an urgent demand for distinguishing HSIL from LSIL. We found the methylation level of *PCDHGB7* in HSIL or CC (defined as “≥HSIL”) was significantly higher than that in LSIL and normal samples (defined as “≤LSIL”) (Figure 2A), implying it could act as a stage divider to classify ≥HSIL from ≤LSIL stage and an early cervical precancerous lesion biomarker. To avoid bisulfite treatment in bisulfite‐PCR pyrosequencing, we modified methylation‐sensitive restriction enzyme combined real‐time fluorescent quantitative PCR (MSRE‐qPCR) to quantify methylation status. In samples with lower methylation levels (10%–20%), the value of ΔCt dropped dramatically (Figure 2B), indicating MSRE‐qPCR was superior for early cancer screening since less cancerous DNA existed alongside relatively lower methylation level. In 404 cervical smears, ΔCt for quantified *PCDHGB7* methylation was significantly lower in ≥HSIL compared with that in ≤LSIL (Figure 2C). Furthermore, the ROC curve showed that MSRE‐qPCR quantification of *PCDHGB7* methylation could be used for classifying CC and distinguishing HSIL from ≤LSIL samples. The AUC was 0.97 for CC, 0.87 for HSIL, and 0.88 for ≥HSIL (Figure 2D). With the methylation cutoff ΔCt = 4.0 when the Youden index is maximized (ΔCt ≤ 4.0 indicates ≥HSIL; ΔCt > 4.0 indicates ≤ LSIL), the specificity was 94.3%, and the sensitivity was 96.0% for CC (Figure 2E).

**FIGURE 2:**
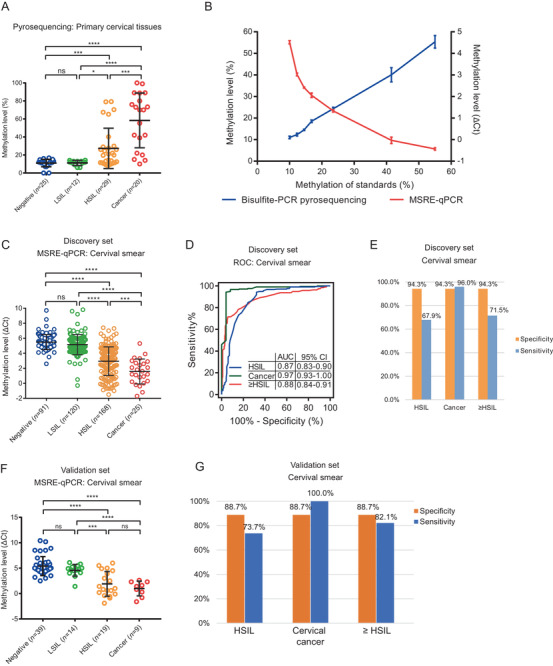
*PCDHGB7* was specifically hypermethylated in cervical cancer and HSIL samples. (A) *PCDHGB7* methylation level was detected by bisulfite‐PCR pyrosequencing in 86 primary cervical tissue samples. (B) The performance of bisulfite‐PCR (BS‐PCR) pyrosequencing and MSRE‐qPCR in detecting selected DNA methylation standard samples. The *x*‐axis indicates the DNA methylation level; seven standard samples were detected; the *y*‐axis in the left indicates the methylation level detected by bisulfite‐PCR pyrosequencing, the *y*‐axis in the right indicates the ΔCt detected by MSRE‐qPCR, and the ΔCt value reflects the DNA methylation. The repeats of pyrosequencing and MSRE‐qPCR were two and three for each grad, respectively. The mean ± SD values were plotted. (C) *PCDHGB7* methylation level of 404 cervical smears in discovery set by MSRE‐qPCR. (D) The ROC curve in 404 cervical smears, and AUC values were illustrated. (E) The sensitivity and specificity of *PCDHGB7* hypermethylation in HSIL, CC, and ≥HSIL group in cervical smears in discovery set. (F) *PCDHGB7* methylation level of 81 cervical smears in validation set by MSRE‐qPCR. (G) The sensitivity and specificity of *PCDHGB7* hypermethylation in HSIL, CC, and ≥HSIL group in cervical smears in validation set. In (A), (C), and (F), error bar represents upper quartile, lower quartile, and median. *P* values were calculated by the unpaired parametric test with GraphPad Prism 7.0. ns, not significant; *, *P *< 0.05; ***, *P *< 0.001; ****, *P *< 0.0001

Next, we comprehensively evaluated the performances of *PCDHGB7* hypermethylation, hrHPV test, and TCT in CC screening (Table 1). For CC, the sensitivity of *PCDHGB7* and hrHPV was similar (96% vs. 95.7%), while the specificity was improved dramatically (94.3% vs. 20.3%). It was also the case in HSIL. As for TCT, its specificity (51.2%) is much lower than that of *PCDHGB7* in CC and HSIL samples. Furthermore, we evaluated the combined effect of *PCDHGB7* hypermethylation, hrHPV test, and TCT. For screening clinical samples with ≥HSIL, if we define “positive” as both positive diagnosis for CC, *PCDHGB7* combined with either hrHPV or TCT increased the specificity to 95.7% and 96.2%, which is higher than either of hrHPV (20.3%) or TCT (51.2%), or the combination of hrHPV and TCT (57.8%). However, the sensitivity of *PCDHGB7* decreased due to these combinations. Similar results were found in three‐method combinations. These results demonstrated that hypermethylated *PCDHGB7* by itself is an ideal alternative tool for CC screening, and there is no need for combining it with either hrPHV test or TCT. Additionally, the robustness of *PCDHGB7* hypermethylation was also testified in the validation set, yielding 82.1% sensitivity and 88.7% specificity for ≥HSIL (Figure 2F); while the sensitivity could reach 100% with 88.7% specificity for identifying CC (Figure 2G).

**TABLE 1:** Performance of *PCDHGB7* methylation detection, hrHPV test, and TCT in early cervical cancer screening

	Negative		LSIL		HSIL						Cervical cancer					
Sample type: cervical smear	Neg/All	Per	Neg/All	Per	Pos/All	Per	Sensitivity	Specificity	PPV	NPV	Pos/All	Per	Sensitivity	Specificity	PPV	NPV
hrHPV Test	31/87	35.6%	9/110	8.2%	155/164	94.5%	94.50%	20.30%	49.70%	81.60%	22/23	95.7%	95.70%	20.30%	12.30%	97.60%
TCT (> = ASCUS)	68/89	76.4%	36/114	31.6%	122/163	74.8%	74.80%	51.20%	55.20%	71.70%	17/23	73.9%	73.90%	51.20%	14.70%	94.50%
DNA methylation	89/91	97.8%	110/120	91.7%	114/168	67.9%	67.90%	94.30%	90.50%	78.70%	24/25	96.0%	96.00%	94.30%	66.70%	99.50%
hrHPV and TCT (> = ASCUS) (any one positive as positive)	27/89	30.3%	2/112	1.8%	163/165	98.8%	98.80%	14.40%	48.70%	93.50%	23/23	100.0%	100.00%	14.40%	11.80%	100.00%
hrHPV and TCT (> = ASCUS) (both two positives as positive)	72/87	82.8%	43/112	38.4%	114/162	70.4%	70.40%	57.80%	57.60%	70.60%	16/23	69.6%	69.60%	57.80%	16.00%	94.30%
DNA methylation and hrHPV (any one positive as positive)	31/87	35.6%	8/112	7.1%	163/166	98.2%	98.20%	19.60%	50.50%	92.90%	25/25	100.0%	100.00%	19.60%	13.50%	100.00%
DNA methylation and hrHPV (both two positives as positive)	89/91	97.8%	111/118	94.1%	106/166	63.9%	63.90%	95.70%	92.20%	76.90%	21/23	91.3%	91.30%	95.70%	70.00%	99.00%
DNA methylation and TCT (> = ASCUS) (any one positive as positive)	67/89	75.3%	34/115	29.6%	153/167	91.6%	91.60%	49.50%	59.80%	87.80%	25/25	100.0%	100.00%	49.50%	19.50%	100.00%
DNA methylation and TCT (> = ASCUS) (both two positives as positive)	90/91	98.9%	112/119	94.1%	83/164	50.6%	50.60%	96.20%	91.20%	71.40%	16/23	69.6%	69.60%	96.20%	66.70%	96.70%
Methylation and TCT (> = ASCUS) and hrHPV (any one positive as positive)	27/89	30.3%	2/114	1.8%	165/167	98.8%	98.80%	14.30%	48.70%	93.50%	25/25	100.0%	100.00%	14.30%	12.60%	100.00%
Methylation and TCT (> = ASCUS) and hrHPV (any two positive as positive)	71/87	81.6%	40/111	36.0%	149/164	90.9%	90.90%	56.10%	63.10%	88.10%	23/23	100.0%	100.00%	56.10%	20.90%	100.00%
Methylation and TCT (> = ASCUS) and hrHPV (all three positive as positive)	90/91	98.9%	6/119	5.0%	77/164	47.0%	47.00%	45.70%	40.30%	52.50%	15/23	65.2%	65.20%	45.70%	11.60%	92.30%

ASCUS, atypical squamous cells of undetermined significance; HSIL, high‐grade squamous intraepithelial lesion; LSIL, low‐grade squamous intraepithelial lesion; NPV, negative predictive values; Per., percentage; Pos, positive; PPV, positive predictive values; TCT, ThinPrep cytology test.

Despite vaginal secretion being much easier to collect than cervical smears, its capacity in CC screening has long been ignored. In 273 vaginal secretions, we found the methylation level of *PCDHGB7* represented by the lowering ΔCt of MSRE‐qPCR was significantly higher in ≥HSIL than in ≤LSIL (Figure 3A). When used for distinguishing patients with CC or HSIL, the AUC were 0.92 and 0.71, respectively (Figure 3B); with 90.4% specificity and 90.9% sensitivity for identifying CC (Figure 3C), these results demonstrated that vaginal secretion is an encouraging sample type for early CC screening by applying *PCDHGB7* methylation detection.

**FIGURE 3:**
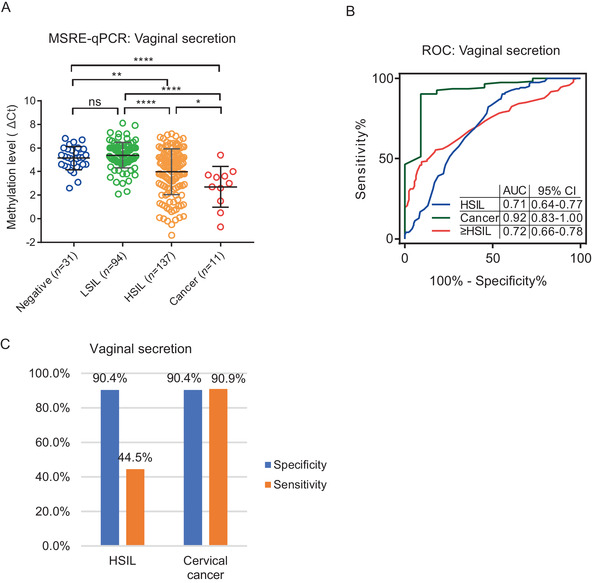
Application of hypermethylated *PCDHGB7* detection for cervical cancer screening by vaginal secretions. (A, B) DNA methylation level (A), and ROC curve (B) in four stages of 273 vaginal secretions. Bars indicate the mean values. *P* values were calculated by the unpaired parametric test with GraphPad Prism 7.0. ns, not significance; **, *P *< 0.05; **, *P* < 0.01; ****, *P *< 0.0001. (C) The sensitivity and specificity of *PCDHGB7* hypermethylation in HSIL and cervical cancer.

Collectively, hypermethylated *PCDHGB7* is identified as a novel UCOM marker and an ideal biomarker for distinguishing HSIL from LSIL. The introduction of *PCDHGB7* makes vaginal secretions feasible for CC screening, which will allow testing to be more easily applied and adopted.

## CONFLICT OF INTEREST

Wenqiang Yu and Shihua Dong report having a pending patent application. The other authors disclosed no potential conflicts of interest.

## ETHICS APPROVAL AND CONSENT TO PARTICIPATE

Samples were collected from Xijing Hospital of Air Force Military Medical University, Jinshan Hospital of Fudan University, and International Peace Maternity and Child Health Hospital. Written informed consent was provided to all patients before sample collection. Institutional Review Board approval for research on human subjects was obtained from hospitals.

## AUTHOR CONTRIBUTIONS

D. S. H., Y. W. Q., and L. Q. designed and initiated the project. D. S. H. and Y. W. Q. supervised the project. D. S. H., X. P., L. Q., C. L. M., D. X. L., M. Z. R., Z. B. L., Y. W. Q., and S. L. generated the data, acquired and managed patients, and provided facilities. D. S. H., X. P., and M. Z. R. performed analysis and interpretation of data. X. P., D. S. H. and Y. W. Q. wrote the manuscript. X. P. and D. S. H. drew the graphical abstract. All the authors read and approved the final manuscript.

## DATA AVAILABILITY STATEMENT

The DNA methylation data are available from UCSC Xena browser (https://xenabrowser.net/), and the expression data are downloaded from TCGA Hub (https://tcga.xenahubs.net). CTCF ChIP‐Seq data were downloaded from ENCODE database.

## Supporting information

Supporting information

TableS1‐S2
