# Supplementary material for: Hypermethylated PCDHGB7 as a universal cancer only marker and its application in early cervical cancer screening
Source: Clin Transl Med. 2021 Jun 20;11(6):e457. doi: 10.1002/ctm2.457 (PMC8214855; doi:10.1002/ctm2.457)
Supplement: Supplementary file 1 — Supporting information [file CTM2-11-e457-s002.docx]

Supplementary Materials for

Hypermethylated *PCDHGB7* as a universal cancer only marker and its application in early cervical cancer screening

Shihua Dong, Qi Lu, Peng Xu, Limei Chen, Xiaoling Duan, Zhanrui Mao, Baolong Zhang, Long Sui, Yudong Wang, Wenqiang Yu

**Correspondence to**: wenqiangyu@fudan.edu.cn (W.Y.), owangydong@126.com (Y.W.), suilong@fudan.edu.cn (L.S.), hathorl@163.com (Q.L.)

**Graphical Abstract**


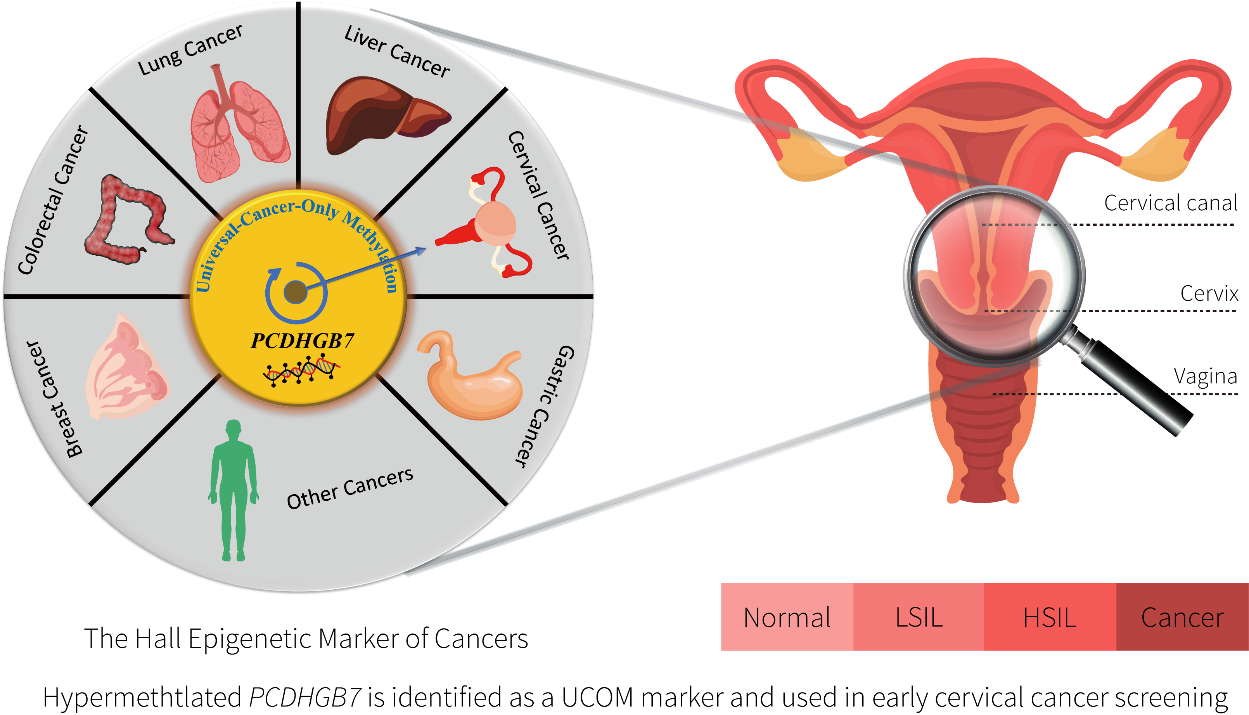


**Materials and Methods**

**TCGA and GEO DNA methylation data analysis**

The Illumina 450K methylation array level 3 data from TCGA database was downloaded from the UCSC Xena browser. The level 3 data has already been normalized and aggregated. The absolute methylation values were calculated from the β values of 450K methylation array [methylation value = (β value + 0.5) ×100%]. Six probes (cg13933262, cg14011639, cg10435816, cg23563234, cg08938584, cg17011276) within the *PCDHGB7* promoter region were selected, the final methylation value was calculated by taking the average of all selected probes. We also downloaded the TCGA expression data from UCSC Xena browser. For each cancer type, when the number of both normal samples and cancer samples was not less than 10, the data was included and analyzed. Clinical information such as histological type, lymphovascular invasion, International Federation of Gynecology and Obstetrics (FIGO) stages, etc., were also obtained from TCGA database. The methylation array data from GEO database (GSE136791, GSE93589, GSE67116, GSE33422, GSE155760, GSE40032) was downloaded (https://www.ncbi.nlm.nih.gov/geo/) and analyzed by the same methods.

Clinical Samples collected for cancer biomarker validation: A total of 727 clinical samples, consisting of 13 cancer types were collected from Xijing Hospital of Air Force Military Medical University and Jinshan Hospital of Fudan University. These samples comprised of 40 biliary cancer (BilC) samples and 14 normal controls, 100 breast cancer (BreC) samples and 50 normal controls; 20 cervical cancer (CerC) samples and 25 normal controls; 31 colorectal cancer (ColC) samples and 16 normal controls; 16 esophagus biliary cancer (EsoC) samples and 16 normal controls; 25 gastric cancer (GasC) samples and 10 normal controls; 11 head and neck cancer (HANC) samples and 10 normal controls; 15 kidney cancer (KidC) samples and 6 normal controls; 25 leukemia (LeuK) samples and 21 normal controls; 29 liver cancer (LivC) samples and 28 normal controls; 25 lung cancer (LunC) samples and 24 normal controls; 22 pancreatic cancer (PanC) samples and 22 normal controls; 84urothelial cancer (UroC) samples and 42 normal controls. Written informed consent was provided to all patients before sample collection. Institutional Review Board approval for research on human subjects was obtained from hospitals.

Clinical Samples of Cervical cancer collected: A total of 844 cervical samples, including 86 formalin-fixed and paraffin-embedded (FFPE) samples, 485 cervical smears (404 in discovery set and 81 in validation set), and 273 vaginal secretions, were collected from Jinshan Hospital of Fudan University and International Peace Maternity and Child Health Hospital, Shanghai Jiaotong University School of Medicine. Note that 86 FFPE samples consist of 25 normal and 20 cervical cancers enrolled in the cancer biomarker study, 12 LSILs, and 29 HSILs. Basic information on the cervical samples was summarized in **Table S2**. For 485 cervical smears, PCR-reverse-dot-blot (RDB) was used for HPV genotyping and 17 types of hrHPV (type 16, 18, 31, 33, 35, 39, 45, 51, 52, 53, 56, 58, 59, 66, 68, 73, and 82) were detected during the screening.

**DNA Extraction**

Genomic DNA (gDNA) was extracted from FFPE samples with TIANquick FFPE DNA Kit (Tiangen Biotech, DP330). gDNA was extracted from cervical smears and vaginal secretions with EP Genomic DNA Kit (Epiprobe Biotech, K-21).

**Bisulfite Pyrosequencing**

A total of 100~200 ng gDNA was taken for bisulfite treatment with EZ DNA Methylation-Gold Kit (ZYMO Research, D5006), and the recovered bisulfite-treated DNA was used as the subsequent PCR template. We detected 11 CpG sites for *PCDHGB7* genomic locus (chr5:141,417,842-141,417,900, GRCh38/hg38). Forward primer 5'-GGTAGAGTGTATTTTTTTTAATTGGAAAAG-3', biotin-labelled reverse primer 5'-biotin-ACAAAAACAACAACAAAAAAAATAATACCTACC-3', and sequencing primer 5'-TTAATTGGAAAAGYGGGGATTTA-3' were used for PCR amplification. The PCR template was amplificated by using 2×Taq PCR Master Mix Kit (Tiangen Biotech, KT201). The PCR program was set as 98 °C 30 s for pre-denaturation, 98 °C 10 s 58 °C 30 s 72 °C 30 for 45-cycle amplification, and 72 °C 3 min for final elongation. The amplified PCR products were confirmed by 2% agarose gel electrophoresis. The pyrosequencing assay was performed on PyroMark Q96 ID (Qiagen) using PyroMark Gold Q96 Reagents (Qiagen, 972804). The methylation level of the *PCDHGB7* promoter region was calculated as the average value of 11 CpG sites. One cytosine in the “CH” was used as bisulfite conversion control in each Pyrosequencing run.

**MSRE-qPCR and DNA Methylation Evaluation**

Methylation-sensitive restriction enzyme combined real-time fluorescent quantitative PCR (MSRE-qPCR) was used for the detection of *PCDHGB7* methylation. There were several cutting sites within the target genomic region. Mechanically, if the CpG dinucleotide within restriction enzymes cutting site was methylated, it cannot be digested; if not, it can be digested but cannot be amplified and detected by subsequent qPCR. Methylation-sensitive restriction enzymes, including HhaI (NEB, R0139) and HpaII (NEB, R0171), were used in MSRE-qPCR. The restriction enzyme cutting sites in the transcription start site (TSS) of *PCDHGB7* were shown in lower case: TCCGAGACccggGACTCCTCCTGTCCTGGGCCGAATGCTCTTTT

AgcgcGGTAGAGTGCACTTTCTCCAACTGGAAAAGCGGGGACCCAGCGAGAACCCGAGCGAACGATGGGAGGGAGCTgcgcGCAGAGgcgccggGccggCCCGCGGCAGGTACTATTTCCTTTGCTGCTGCCTTTGTTCTACCCCACGCTGTGTGAGCCGATCCGCTACTCGATTccggAGGAGCTGG.The *GAPDH* gene-related region absent of any cutting sites was selected for normalization. For each digestion reaction, 100 ng gDNA was taken as input, and every two units of the endonucleases mentioned above were added, making the final volume to be 25 μl, followed by digestion at 37 °C for 30 min and heat inactivation at 95 °C for 5 min. TaqMan probe and primers designed for *PCDHGB7* and *GAPDH* were as follow: for *PCDHGB7*, forward primer 5'-TTCTCCAACTGGAAAAGC-3', reverse primer 5'-AGCAGCAAAGGAAATAGTA-3', and probe 5'-VIC-CGAGAACCCGAGCGAACG-MGB-3'; for *GAPDH*, forward primer 5'-GTCCTTGACTCCCTAGTG-3', reverse primer 5'-GGGAAGGGACTGAGATTG-3', and probe 5'-CY5-TGCCCACAGTCCAGTCCT-MGB-3'. The subsequent dual real-time PCR was performed on 7500 Real-Time PCR System (Life Technologies) with a program as follows: initiation at 95 °C for 10 min; then 45 cycles of 94 °C for 20 s, 60 °C for 60 s. The DNA methylation level for each sample was evaluated by ΔCt = Ct_*_PCDHGB7_* – Ct_*_GAPDH_*.

For methylation standard curve, we acquired genomic DNA (gDNA) from human white blood cells (WBCs) and HeLa cells, the *PCDHGB7* methylation level of which were 10% (low) and 55% (high), respectively. The WBC gDNA was incorporated by 0%, 5%, 10%, 25%, 50%, 75%, and 100% Hela gDNA. Thus, the methylation level=10% (1-*x*) + 55%*x* (where *x* indicates the Hela gDNA incorporation ratio), and the resulting methylation gradients were 10.00%, 12.25%, 14.50%, 16.75%, 23.50%, 41.50%, and 55.00%.

**Statistical analysis**

Receiver operating characteristic (ROC) curve was constructed to quantify the diagnostic performance of hypermethylated *PCDHGB7* by assessing its sensitivity, specificity, and respective areas under curve (AUC) with 95% confidence interval (CI) in a cervical cancer classifier system. The significance of differences was evaluated with two-tailed unpaired parametric test. *P*-values less than 0.05 were considered significant (*, *P* < 0.05; **, *P* < 0.01; ***, *P* < 0.001; ****, *P* < 0.0001). Error bar represents upper quartile, lower quartile, and median. All statistical analyses were performed using GraphPad Prism 7.0.

**Supplementary Figures and Tables**


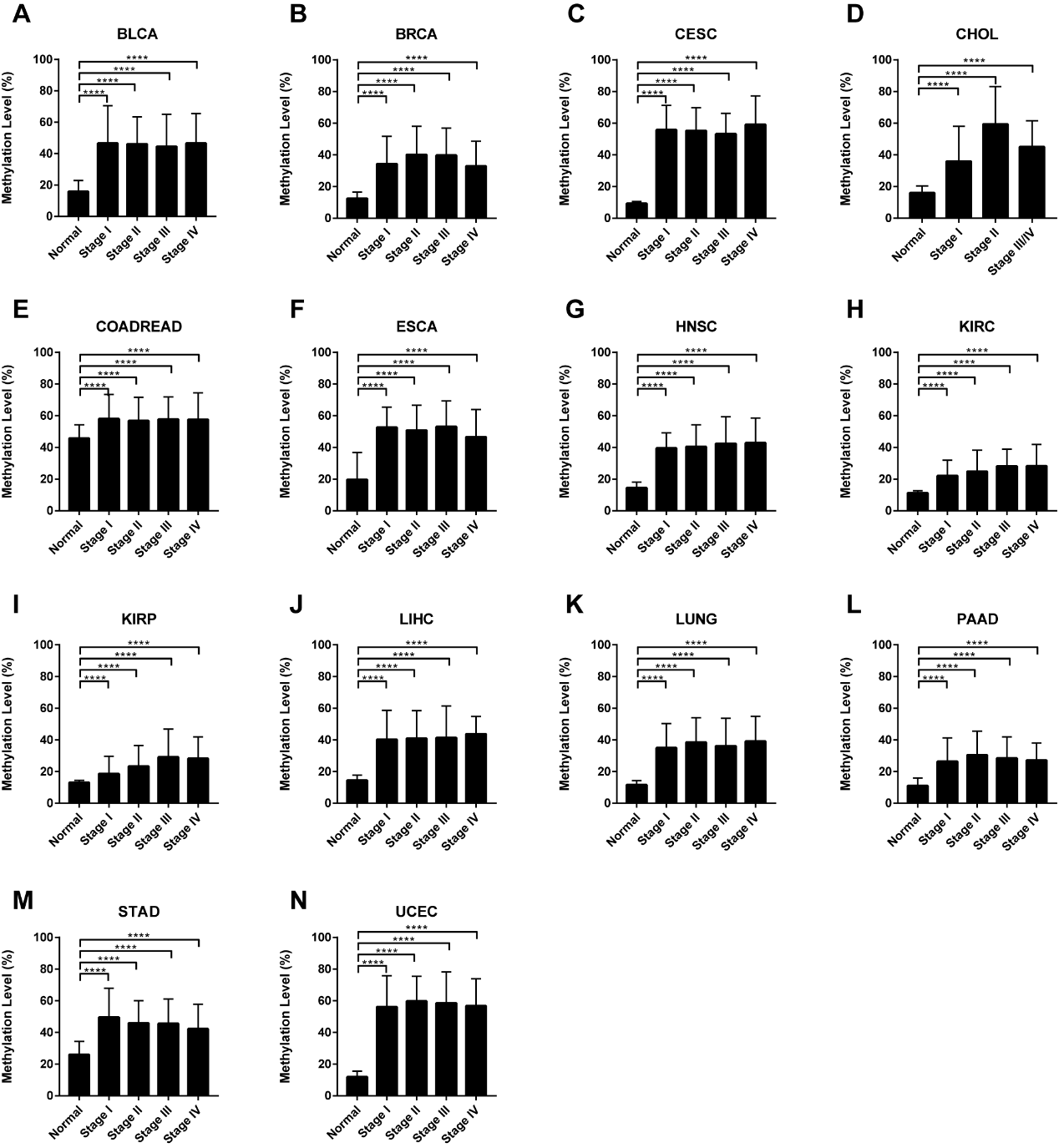


**Figure S1. *PCDHGB7* methylation in different stages of cancer in 14 TCGA cohorts.** BLCA, bladder urothelial carcinoma; BRCA, breast invasive carcinoma; CESC, cervical squamous cell carcinoma and endocervical adenocarcinoma; CHOL, cholangiocarcinoma; COADREAD, colon adenocarcinoma and rectal adenocarcinoma; ESCA, esophageal carcinoma; HNSC, head and neck squamous cell carcinoma; KIRC, kidney renal clear cell carcinoma; KIRP, kidney renal papillary cell carcinoma; LIHC, liver hepatocellular carcinoma; LUAD, lung adenocarcinoma; PAAD, pancreatic adenocarcinoma; STAD, stomach adenocarcinoma; UCEC, uterine corpus endometrial carcinoma. *P*-values were calculated using the two-tailed unpaired parametric test by GraphPad Prism 7.0. ****, *P* < 0.0001.


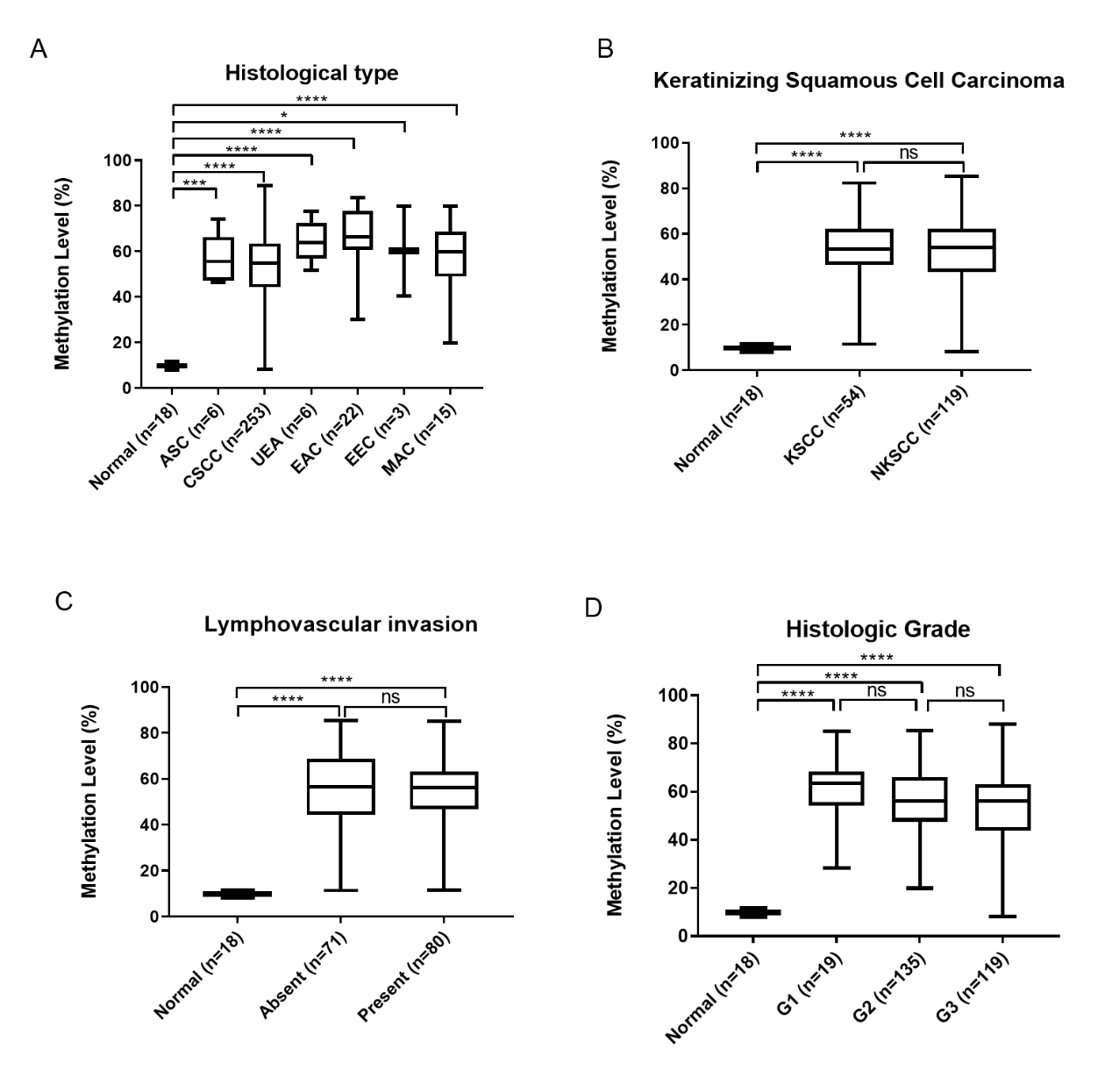


**Figure S2. *PCDHGB7* methylation level in TCGA CESC cohort and GEO datasets by different classification strategies.** ASC, Adenosquamous carcinoma; CSCC, Cervical Squamous Cell Carcinoma; UEA, Endocervical Adenocarcinoma of the Usual Type; EAC, Endocervical Type of Adenocarcinoma; EEC, Endometrioid Adenocarcinoma of Endocervix; MAC, Mucinous Adenocarcinoma of Endocervical type; KSCC, keratinizing squamous cell carcinoma; NKSCC, non-keratinizing squamous cell carcinoma. *P*-values were calculated using the two-tailed unpaired parametric test by GraphPad Prism 7.0. *, *P* < 0.05; ****, *P* < 0.0001; ns, not significant.


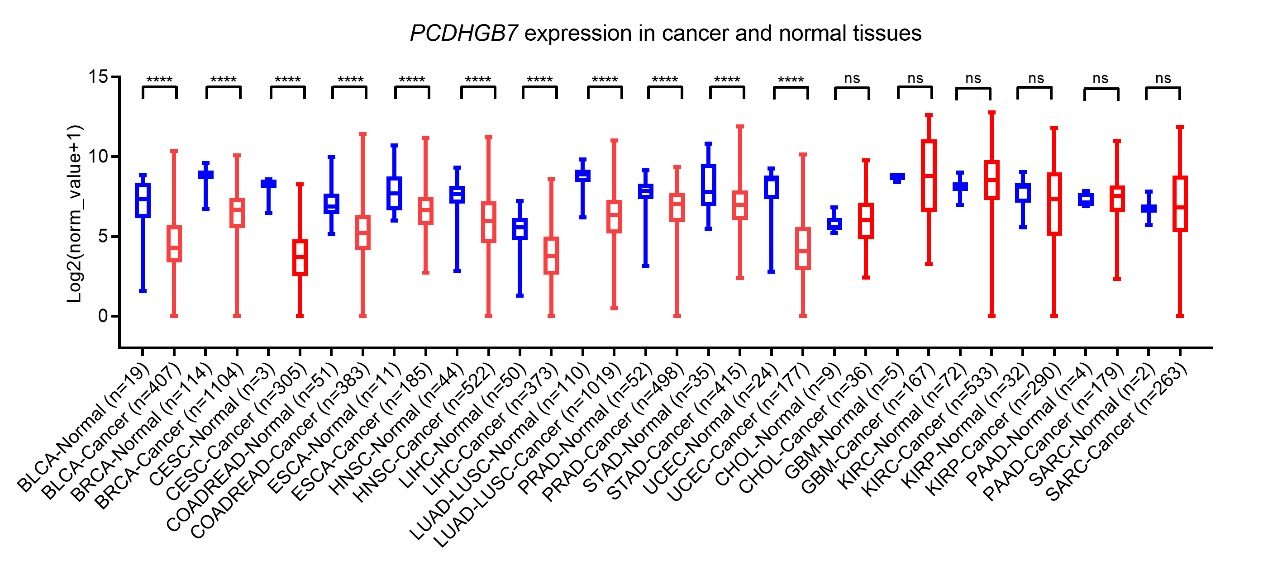


**Figure S3. *PCDHGB7* expression level in 17 types of cancer and normal tissues.** BLCA, bladder urothelial carcinoma; BRCA, breast invasive carcinoma; CESC, cervical squamous cell carcinoma and endocervical adenocarcinoma; CHOL, cholangiocarcinoma; COADREAD, colon adenocarcinoma and rectal adenocarcinoma; ESCA, esophageal carcinoma; GBM, glioblastoma multiforme; HNSC, head and neck squamous cell carcinoma; KIRC, kidney renal clear cell carcinoma; KIRP, kidney renal papillary cell carcinoma; LIHC, liver hepatocellular carcinoma; LUAD-LUSC, lung adenocarcinoma and lung squamous cell carcinoma; PAAD, pancreatic adenocarcinoma; PRAD, prostate adenocarcinoma; SARC, sarcoma; STAD, stomach adenocarcinoma; UCEC, uterine corpus endometrial carcinoma. Data was download from TCGA database. *P*-values were calculated using the two-tailed unpaired parametric test by GraphPad Prism 7.0. ****, *P* < 0.0001; ns, not significant.


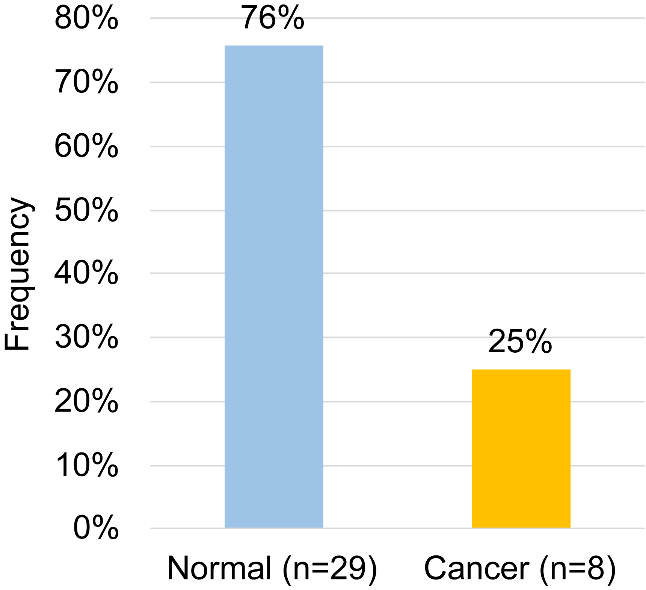


**Figure S4. CTCF binding frequency in *PCDHGB7* promoter region in normal and cancer samples.** CTCF ChIP-Seq data in eight cancer types and 29 normal tissue samples were downloaded from ENCODE database. *P*-value < 0.05, two-tailed student’s t-test.

**Table S1.** The AUC of *PCDHGB7* hypermethylation as a biomarker in 13 cancer types

**Table S2.** The basic information and DNA methylation value of 844 cervical samples
